# Eleven Letters with Answers

**Published:** 1875-07

**Authors:** 


### Our Correspondence.

### Letters Exposing Humbugs and Swindlers,

**With other Communications of Interest to the People.**

SUNBURY, Pa., June 10, 1875.

ED. BISTOURY :

Where can I procure such an apparatus for over-riding toes as you describe in last number of the BISTOURY ? S. M.

Of Geo. Tiemann & Co., 67 Chatham St., New York, or at this office.

---

TROY, ALA., June 16, 1875.

DR. UP DE GRAFF :

Please send me copy of American Agriculturist and the BISTOURY. I would like to see them as I have heard so much about them. J. F. R.

We cannot send specimen copies of other journals, you must apply to the publishers.

---

BRUNSWICK, MO., June 14th, 1875.

DR. THAD S. UP DE GRAFF :

*Dear Sir:*—Enclosed find price of BISTOURY for another year. I am exceedingly well pleased with your journal, and wish it were a monthly instead of a quarterly. J. S. W., M. D.

So say many of our friends, Doctor, and some day we may gratify you.

---

JAMESTOWN, N. Y., June 12, 1875.

EDITOR BISTOURY :

I see advertisements of the "Howard Association," who agree to cure certain nervous ailments for a specified sum of money. My son has been patronizing them for some time, has paid them much money, also a concern called the "Peabody Institute," of Boston. I begin to fear they are not what they should be. Can you give me any information about them? J. S. B.

We are surprised that a man who has arrived at the years of discretion should allow himself to be swindled by any of these "manhood" fellows.—The "Associations" are managed by one man each, whose occupations are to catch a greenhorn and relieve him of his money whenever and wherever they can find him. It seems they discovered one in Jamestown. If you have any money left, send your son to some respectable physician in your own town and leave these New York advertising charlatans alone. Spank your son and tell him he should have known better, then let him do the same by you.

DR. UP DE GRAFF.

Am dropping several of my medical journals, for which I have no leisure time—not wishing to pay for what is no worth to me. The BISTOURY costs little, is not hard reading, can be caught up when on journeys to my patients, is racy and spicy, and always has something valuable ; in fact I cut out most of the prescriptions and paste them into my pocket memorandum book.

The above is an opinion of the merits of the BISTOURY, by an eminent eastern physician.

---

NEOSHO, MO., May 10, 1875.

DR. UP DE GRAFF :

Please inform me in your July number of the BISTOURY, whether there is any preventive for moles coming upon the face, and can they be removed when quite small? Any information concerning them will be thankfully received by a constant reader. C. L. M.

There is no means known for preventing the formation of moles. They can be completely and almost painlessly removed by means of the electro-cautery.

---

THOMASVILLE P. O. CHEATHAM, CO., TENN., May 9th, 1875.

THAD S. UP DE GRAFF, M. D. :

*My Dear Sir.*—I have a friend who is troubled with incessant itching of the scalp, pustules forming, then, after discharging their contents, form small sores, which scab over and become perfectly dry. Prescription after prescription, by numerous physicians, have been applied but no benefit. I want you to send me a ℞. through the columns of the BISTOURY. T. W. W.

℞

Cupri sulphas, gr. xxx.
Ceratum simplex, ℥ ss.

Pulverize the cuprum until reduced to an impalpable powder, then mix it thoroughly with the cerate. Wash the scalp with castile soap and warm water, removing all the scabs. Dry with a soft cloth, then rub the ointment into the scalp. The hair must be cut short and a black silk cap worn on the head. Make the application twice daily, morning and evening. Give internally, the following, three times a day, in half teaspoonful doses, immediately after meals:

℞

Vini ferri, ℥ jss.
Syrupi simplicis.
Liquoris arsenicalis, āā ʒ ij.
Aquæ destillatæ, ℥ ij. Misce.

MONTOURSVILLE, PA., May 26, 1875.

DR UP DE GRAFF:

I see in the April number of the BISTOURY an article and an illustration of a disease of the eye-lids which you call *ectropium*. I think my father, aged 80, has this identical disease. Do you think he is too old to be operated upon? His health is pretty good, but his eye-lids are so sore and inflamed that they give him much trouble. If you think it safe, we will bring him to you for an operation. J. R. T.

Your father is not too old to undergo such an operation. It will give him great relief to have the lids restored to their proper position.

---

IPSWICH, Mass., June 6, 1875.

DR. UP DE GRAFF:

I see your BISTOURY here, and have read your answers to correspondents. I think I have liver-complaint, and I have taken six bottles of Kennedy's Discovery without much benefit. Can you recommend anything better to take than that?

J. R. M.

Yes, certainly; let's see—why, take Costar's Rat Exterminator—Piso's Bed Bug Poison—Rising Sun Stove Polish—Centaur Liniment, said to be good for man or beast—Cincho's Quinine, or any other advertised nostrum. They are all good for something, and that something is as likely to be "liver complaint" as anything else. Try them all, and, if you are alive then, report your success.

---

OLUSTEE CREEK, PIKE CO., ALA.,
June 22, 1875.

DR. THAD S. UP DE GRAFF:

*Dear Sir*—Ever since I first heard of your skill, and success, I have intended writing you in reference to my little daughter who is sadly afflicted, being almost deprived of both sight and hearing. We think the cause is scrofula. She has been a great sufferer all her life. When she was an infant, the first cause of her suffering that we could locate, was an abscess in her ear. Then she had something like neuralgia in her eye. Afterward she suffered greatly with pains in her limbs, and all over her body. The physicians treated her for rheumatism. It was two years before she was free from pains. She was so emaciated we could see no signs of life, except by a faint breathing. When she was four years old, her sight began to fail. Her eyes were red, bloodshot, and very much inflamed, for a week. As that disappeared, there came a white mist or film over them, and she went totally blind for three months. Her eyes have a dull, glassy look, with white spots below the sight, extending across the eyeballs. They do not overflow with tears as they did at first. She can distinguish some bright colored objects, when held *very near* her eyes. Three years ago, she had polypus in the nose. That has been well for some time, until recently, we discovered some symptoms of its return. She is now nearly fourteen years of age, and has enjoyed excellent health, for the *past two* years, except occasional severe headaches, or neuralgia, for which we administered large doses of quinine, which always gave temporary relief. But saddest of all, and more to be regretted than all her afflictions, she is almost deaf. Her hearing is so much impaired already, she can hear only when we speak in loud tones, and is daily *growing worse*. Please write by return mail and give me your opinion of the case. If it is hopeless, do not hesitate to tell me so.

Yours respectfully, J. A. D.

We give the above letter almost entire, partly to show our readers that we are only really miserable through comparison.

The child is evidently scrofulous, and is blind from opacity of the cornea. Should there exist a clear spot upon either cornea, large enough to permit the formation of an artificial pupil, partial vision could be restored. The deafness, likely, is produced from the administration of quinine. The child should be sent to an oculist for examination.

---

OWEGO, N. Y., June 20, 1875.

DR. UP DE GRAFF:

A man came through our town recently, selling spectacles, and said he was your agent. He sold me a pair of spectacles for $4.50, and said I could send them to you and get my money back if they did not fit. I showed them to our jeweler here, and he says he would like to sell me all I can carry of such spectacles for a dollar a pair. He also said he thought the fellow was a swindler, and that you do not sell spectacles. I write to find out if this is so. * * * *

R. B.

Your jeweler is right—you are swindled. We do not deal in spectacles, and if we did, we should not hawk them from door to door, through the agency of a fourth-rate peddler. Is it not strange that people prefer to patronize a traveling humbug to one of their own townsmen, and pay ten prices for the privilege of being bamboozled? We think so.

